# Very Low-Calorie Ketogenic Diet (VLCKD) as Pre-Operative First-Line Dietary Therapy in Patients with Obesity Who Are Candidates for Bariatric Surgery

**DOI:** 10.3390/nu15081907

**Published:** 2023-04-14

**Authors:** Luigi Barrea, Ludovica Verde, Luigi Schiavo, Gerardo Sarno, Elisabetta Camajani, Antonio Iannelli, Massimiliano Caprio, Vincenzo Pilone, Annamaria Colao, Giovanna Muscogiuri

**Affiliations:** 1 Dipartimento di Scienze Umanistiche, Università Telematica Pegaso, Centro Direzionale, Via Porzio, Isola F2, 80143 Naples, Italy; 2 Centro Italiano per la cura e il Benessere del Paziente con Obesità (C.I.B.O), Unità di Endocrinologia, Diabetologia e Andrologia, Dipartimento di Medicina Clinica e Chirurgia, Università degli Studi di Napoli Federico II, Via Sergio Pansini 5, 80131 Naples, Italy; 3 Department of Public Health, University of Naples Federico II, Via Sergio Pansini 5, 80131 Naples, Italy; 4 Department of Medicine, Surgery and Dentistry “Scuola Medica Salernitana”, Complex Operative Unit of General and Emergency Surgery and Bariatric Centre of Excellence SICOB, University of Salerno, 84081 Salerno, Italy; 5 San Giovanni di Dio e Ruggi D’Aragona University Hospital, Scuola Medica Salernitana, 84131 Salerno, Italy; 6 Department of Human Sciences and Promotion of the Quality of Life, San Raffaele Open University, 00166 Rome, Italy; 7 Centre Hospitalier Universitaire de Nice-Digestive Surgery and Liver Transplantation Unit, Archet 2 Hospital, 151 Route Saint Antoine de Ginestière, BP 3079, CEDEX 3, 06200 Nice, France; 8 Faculté de Medicine, Université Côte d’Azur, 06000 Nice, France; 9 Inserm, U1065, Team 8 “Hepatic Complications of Obesity and Alcohol”, 06204 Nice, France; 10 Laboratory of Cardiovascular Endocrinology, Istituto di Ricovero e Cura a Carattere Scientifico (IRCCS) San Raffaele, 00166 Rome, Italy; 11 Unità di Endocrinologia, Diabetologia e Andrologia, Dipartimento di Medicina Clinica e Chirurgia, Università degli Studi di Napoli Federico II, Via Sergio Pansini 5, 80131 Naples, Italy; 12 Cattedra Unesco “Educazione Alla Salute e Allo Sviluppo Sostenibile”, Università degli Studi di Napoli Federico II, Via Sergio Pansini 5, 80131 Naples, Italy

**Keywords:** ketogenic diet, very low-calorie ketogenic diet, obesity, bariatric surgery, nutrition

## Abstract

Bariatric surgery is currently the most effective method for achieving long-term weight loss and reducing the risk of comorbidities and mortality in individuals with severe obesity. The pre-operative diet is an important factor in determining patients’ suitability for surgery, as well as their post-operative outcomes and success in achieving weight loss. Therefore, the nutritional management of bariatric patients requires specialized expertise. Very low-calorie diets and intragastric balloon placement have already been studied and shown to be effective in promoting pre-operative weight loss. In addition, the very low-calorie ketogenic diet has a well-established role in the treatment of obesity and type 2 diabetes mellitus, but its potential role as a pre-operative dietary treatment prior to bariatric surgery has received less attention. Thus, this article will provide a brief overview of the current evidence on the very low-calorie ketogenic diet as a pre-operative dietary treatment in patients with obesity who are candidates for bariatric surgery.

## 1. Introduction

Obesity is a growing concern worldwide, with significant health and economic consequences. This chronic condition is associated with increased risk of mortality [1] and a range of health problems including hypertension, dyslipidaemia, type 2 diabetes mellitus (T2DM), cardiovascular disease, and several types of cancers [2]. Bariatric surgery (BS) has emerged as a definitive treatment for obesity and its related complications [3,4]. In fact, BS is the most effective treatment for patients with severe obesity in terms of permanent weight loss and the reduction of comorbidity and mortality [3,4].

Among the various surgical techniques, Roux-en-Y gastric bypass (RYGB) and sleeve gastrectomy (SG) are the most commonly used [5], and they are usually performed laparoscopically [6]. However, laparoscopic surgery in patients with obesity can be challenging, as the thickness of the abdominal wall, the accumulation of visceral adipose tissue, and an enlarged liver volume can obstruct the surgical field [7] and increase the risk of complications, such as anastomotic leakage, bleeding, and infection [8]. Excess visceral fat can increase the risk of surgical complications and prolong the conversion rate and surgical time [8]. For instance, an enlarged liver and the accumulation of visceral fat can obstruct the surgical field, which is responsible for approximately 50% of conversion cases in RYGB [9]. Additionally, a large neck circumference (>44 cm) may lead to difficulties in intubation and mechanical ventilation [10]. Immediate preoperative weight loss has been reported to reduce anaesthesiological and surgical risks [11] and improve short- and long-term outcomes [12], but its role is still a subject of debate [13]. The evidence on the effects of preoperative weight loss comes mainly from retrospective studies, as there is a lack of multicentre randomized controlled trials (RCTs) on this specific topic [14]. Therefore, guidelines do not currently provide conclusive evidence for preoperative weight loss [15,16].

Several approaches to weight loss before surgery have been explored, including pharmacotherapy with glucagon-like peptide-1 receptor agonists [17] or a hypocaloric diet combined with gastric balloon insertion [18]. These methods appear to be effective in reducing the rate of surgical complications and conversions [19].

However, in clinical practice, diet is probably the most common approach. There are various diets that can be recommended to patients undergoing BS, with studies showing that there are a wide range of diets prescribed across different centres [20,21]. These diets include low-calorie (LCD), low-carbohydrate, and liquid-based diets [20,21].

The Mediterranean diet, which mainly consists of plant-based foods and uses olive oil as the primary source of added fat, has been linked to numerous health advantages, such as a decreased risk of chronic diseases [2]. However, research indicates that it may not be the most efficient method for rapid weight loss prior to BS [22]. By adopting a balanced, energy-controlled diet before the operation, similar to the Mediterranean diet, patients can improve their nutritional habits and enhance their nutritional status [23]. However, it may not be restrictive enough to help reduce body weight significantly and quickly before BS [22]. According to some authors, patients who follow a diet more strictly before surgery tend to lose more weight after surgery, for example, with an LCD [24]. For high-risk patients, a very low-calorie diet (VLCD), which involves consuming 600–800 kcal per day, may be a viable option to achieve rapid weight loss [25]. However, LCDs have certain disadvantages, which are the greater the more restrictive they are, such as the loss of lean mass; poor nutrient intake, if not well supplemented; and difficult diet adherence.

Research has shown that low-carbohydrate diets can help reduce liver fat and volume [26,27], which may be beneficial for patients undergoing BS. While personalized diets are generally more effective in promoting adherence, standardized diets may be more appropriate in preparing patients for surgery in a short period of time. In this regard, although they do not promote sustainable changes in eating habits, ketogenic diets (KDs) may be more effective as pre-operative diets for BS, while diets along the Mediterranean lines may better serve as post-operative diets for maintaining the weight loss.

KD is a term that refers to various low-carbohydrate diet protocols. These diets are characterized by a high intake of fats and proteins, resulting in a fasting-like state that promotes physiological ketosis [28]. For instance, the very low-calorie ketogenic diet (VLCKD) involves a significant reduction in carbohydrate consumption (less than 50 g per day), adequate protein intake, and high fat consumption, with an average energy intake of 800 kcal/day [28,29,30]. While the KD was originally used to treat epilepsy in children [31], it has been shown to be an effective means of inducing rapid weight loss and managing obesity-related disorders in adults [32,33,34,35]. Recent research has demonstrated that the VLCKD may be a particularly attractive pre-operative dietary treatment for patients with obesity who are candidates for bariatric surgery. In fact, a recent RCT found that VLCKD resulted in better surgical outcomes than a VLCD in 178 patients undergoing laparoscopic SG [36].

Overall, KD has been shown to be an effective strategy for inducing rapid weight loss, and its use before surgery, especially when available in the short term, is particularly attractive [37]. The aim of this review was to summarize the current evidence on the VLCKD as pre-operative dietary treatment in patients with obesity candidates for BS.

## 2. Very Low-Calorie Ketogenic Diet

### 2.1. Definition of Ketogenic Diets

KDs are high-fat diets, characterized by a carbohydrate restriction (30–50 g per day) [28]. This drastic reduction in the content of exogenous carbohydrates drives the body into a state of mild physiological ketosis: a metabolic state characterised by an increase in the concentration of ketone bodies [38]. Ketone bodies are the products of hepatic ketogenesis, namely acetoacetate, acetone, and β-hydroxybutyrate (although the latter is not defined as a ketone by IUPAC nomenclature) [38]. Various KD protocols exist, differing from each other based on calories, macronutrients composition, and the achievable ketogenic ratio [28]. The term ketogenic ratio refers to the ratio between the amount of lipids (expressed in grams) in the diet protocol and the amount of protein and carbohydrates [28].

The most used KD therapies in the treatment of obesity are the low-calorie ketogenic diet (LCKD) and the VLCKD. These nutritional approaches exploit nutritional ketosis, induced not only by low carbohydrate intake but also by calorie restriction, to achieve a rapid loss of fat mass while preserving lean mass [39]. Recently, VLCKD has been shown to result in significant weight loss along with improved glycaemic control in subjects with obesity and T2DM [40,41,42]. The VLCKD protocol is characterized by a daily calorie diet of 700–800 kcal/day with a carbohydrate restriction of 30–50 g/day (≃13% of total energy intake), a 30–40 g/day (≃44%) increase in fats, and about 1.2–1.4 g/day proteins per kg body weight (≃43%) [29,30]. While some may mistakenly believe that VLCKD is a high-protein diet, it actually maintains a daily protein intake of around 1.2–1.5 g/kg of ideal body weight. Furthermore, VLCKD is based on high-quality protein sources from both animal and non-animal sources, such as eggs, peas, soy, and whey protein [29,30].

### 2.2. Mechanisms of Action and Benefits of Very Low-Calorie Ketogenic Diet before Bariatric Surgery

As reported in the Italian Society of Obesity Surgery and Metabolic Diseases (SICOB) guidelines, the pre-operative reduction of body weight is recommended in patients who are candidates for BS, especially in the presence of BMI > 40 kg/m^2^ or severe visceral obesity, including the prescription of a LCD/KD in the pre-operative period [43]. Decreasing body weight significantly reduces visceral adipose tissue and fat liver [44], facilitating the performance of laparoscopic operations, reducing the performance time and the risk of conversion [9], and improving short- and long-term results, especially in patients with BMI > 40 kg/m^2^ [45,46].

Several methods have been proposed to promote preoperative weight loss. In a prospective observational study, Colles et al. investigated the efficacy and acceptability of a preoperative very low energy diet (VLED) [47].

In a study involving 32 participants (19 men and 13 women) with a mean BMI of 47.3 ± 5.3 kg/m^2^, a VLED was implemented for 12 weeks. The study aimed to measure changes in liver volume, visceral and subcutaneous adipose tissue, body weight, anthropometric measures, and biochemical variables. Compliance, acceptability, and side effects were also evaluated. The study found that the degree of liver volume reduction was directly related to the reduction in relative body weight and initial liver volume. Eighty percent of the reduction in liver volume occurred between weeks 0 and 2. Reductions in body weight and visceral adipose tissue were consistent over the 12-week period. Based on these findings, the authors suggest that a pre-operative VLED should be followed for a minimum of 2 weeks to achieve reductions in liver volume and visceral adipose tissue. Ideally, a 6-week duration would be best to achieve maximal liver volume reduction and significant reductions in visceral adipose tissue and body weight without affecting compliance or acceptability [47]. Leonetti et al. enrolled 50 patients (31 females and 19 males, mean age 47.7 ± 11.2 years, mean BMI 53.5 ± 8.4 kg/m^2^) who followed a VLCKD and VLCD protocol prior to BS treatment (the obese preoperative diet (OPOD) group) and were compared with 30 patients (18 females and 12 males, mean age 43.3 ± 8.7 years, mean BMI 54.8 ± 9.4 kg/m^2^), who followed a standard LCD (control group) [48]. Body weight and waist circumference decreased significantly in the OPOD group, whereas no significant changes occurred in the control group. The OPOD group also recorded an improvement in fasting plasma glucose levels, even in patients with T2DM taking antidiabetic drugs. No significant changes were found in plasma creatinine, urea, uric acid, glutamic oxaloacetic transaminase, glutamic pyruvic transaminase, γ-glutamyl transferase, or alkaline phosphatase levels, confirming the liver and kidney safety of this protocol. An ultrasound evaluation was performed and an average 30% reduction in liver volume was found [48].

From the evidence in the literature, it seems clear that the use of a VLCD or VLCKD in the 15 to 30 days prior to surgery achieves satisfactory results in less time, at a lower cost, and with fewer side effects than the intragastric balloon [18,49].

According to Albanese et al., the main advantage of VLCKD is not only fast and substantial weight loss but also its positive influence on parameters strongly related to surgical outcome [36]. In fact, in a recent study of 178 patients who underwent either VLCKD or VLCD before SG, blood drainage outputs were lower and post-operative haemoglobin levels were higher in the group following VLCKD than the group following VLCD. Considering that weight loss and mean operative time were comparable between the two groups, it can be assumed that this advantage was also influenced by the greater ease of surgical manoeuvres due to hepatomegaly and visceral adipose tissue reduction. The authors surmised that patients with VLCKD achieved a better metabolic and nutritional status that influenced tissue healing and response to surgery [36]. In line with these results, a 4-week preoperative VLCKD that included micronutrient supplementation led to better blood glucose and hypertension, as well as a 19.8% decrease in the initial volume of the left hepatic lobe [50].

Another important benefit of VLCKD is the high compliance rate due to the anorexigenic effect and hunger reduction caused by ketone bodies [51]. When the body is in a state of ketosis, it uses ketone bodies as a primary source of energy instead of carbohydrates. This shift in metabolism can lead to decreased hunger and cravings, making it easier for patients to stick to the prescribed diet [51]. In addition, the physiological production of beta-hydroxybutyrate during VLCKD exerts an important anticatabolic effect on skeletal muscle, thus leading to a decrease in fat mass, preserving lean mass and muscle strength [52].

For this reason, the Italian Society of Endocrinology (SIE) Consensus Statement recommends a 2- to 6-week preoperative weight-loss program with VLCKD for patients who are candidates for BS in order to induce weight loss and a reduction in liver volume and visceral adipose tissue [40].

**Figure 1:**
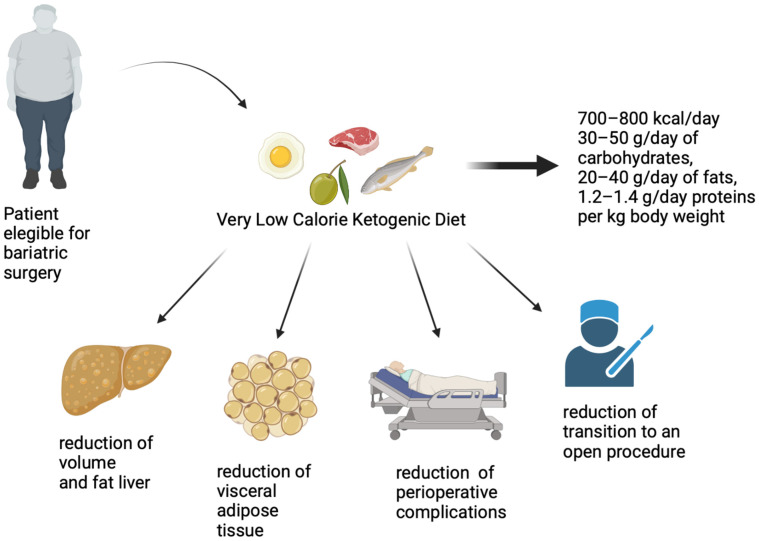
Pre-operative effects of very low-calorie ketogenic diet in a candidate for bariatric surgery.

Therefore, VLCKD is effective in rapidly reducing weight, waist circumference, and liver volume and consequently reduces the risk of transitioning to an open procedure, as well as the risk of perioperative complications (Figure 1).

### 2.3. Indications and Contraindications of Very Low-Calorie Ketogenic Diet in Pre-Bariatric Surgery

According to Marinari et al., losing weight before surgery can decrease liver volume and potentially make the surgery easier [53]. However, there is still debate over whether weight loss before surgery reduces the risk of complications after surgery [16,54].

As stated in the European Association for Endoscopic Surgery (EAES) clinical practice guidelines on BS (2020) endorsed by the European Association for the Study of Obesity (EASO), the International Federation for the Surgery of Obesity and Metabolic Disorders (IFSO-EC) and the European Society for the Peri-operative Care of the Obese Patient (ESPCOP), three RCTs were found regarding preoperative diet consultation versus standard care in patients undergoing BS [54]. The results showed that the group that received preoperative diet consultation had more significant weight loss after surgery (SMD 0.4, 95% CI 0.03 to 0.78 higher), but there was no significant difference in the likelihood of postoperative complications (risk ratio, RR, 0.80, 95% CI 0.22 to 2.86), although interval estimates were wide [54]. However, a study from a Swedish registry showed a decrease in complications after gastric bypass surgery [55].

Moreover, as reported by Marinari et al., it is necessary to improve the preoperative fasting blood glucose level by using diet, exercise, and medication [55]. This is because having a blood glucose level higher than 180 mg/dl has been linked to an increase in complications and mortality during the surgery [56]. Considering these assumptions, it would seem evident that a VLCKD is effective in the rapid loss of visceral adipose tissue and hepatic adipose tissue prior to BS, thus aiding the surgery.

According to the Position Statement of SIE, VLCKD should be stopped 48 h prior to elective surgery or invasive procedures and perioperative period [40]. On the other hand, there are several absolute contraindications regarding the use of VLCKD, such as type 1 diabetes mellitus, latent autoimmune diabetes in adults, β-cell failure in T2DM, the use of sodium/glucose cotransporter 2 inhibitors, kidney failure, moderate-to-severe chronic kidney disease, liver failure, hearth failure (NYHA III-IV), and respiratory failure [29,30].

### 2.4. Side Effects and Transient Complications of Very Low-Calorie Ketogenic Diet

VLCKD is a dietary treatment that may have transient adverse effects in the short to medium term. One of the most frequent complications of the VLCKD nutritional program is dehydration, and for this reason, an intake of 2–2.5 L of water or other sugar-free beverages daily is recommended, especially during the active phase of ketosis (PMID: 35653127). Dehydration can lead to electrolyte disorders, such as hyponatremia, hypokalemia, and hypomagnesemia (PMID: 31665015). As reported by Barrea et al. (PMID: 35653127), these disorders can also develop due to the urinary excretion of ketone bodies and low micronutrient intake, especially during the active phase. Therefore, proper hydration and supplementation with vitamins and minerals, as reported by the European Food Safety Authority (EFSA), is essential.

Another complication that may occur in the short to medium term is increased uricemia. In a study by Bruci and colleagues, uric acid was finally shown to be significantly reduced after a 90-day VLCKD protocol, ruling out a correlation between VLCKD and hyperuricemia (PMID: 32012661). A meta-analysis conducted by Castellana et al. reported an overall neutral effect on uric acid by VLCKDs (PMID: 31705259). These controversial results could be explained by the timing and extent of weight loss, as food groups that typically increase serum uric acid levels (including red meat and anchovies) are widely consumed in KDs and could lead to this effect in the short term (PMID: 32012661). However, it is recognized that weight loss is associated with a significant reduction in urate levels (PMID: 31468681). For this reason, it seems reasonable to suggest that it is necessary to monitor uricemia throughout the course of VLCKD, and, in the case of patients with hyperglycaemia, limit foods with high urate content and administer allopurinol if necessary.

Another complication that could occur due to lower food and fibre intake is constipation, which responds well to sufficient fluid intake, the daily intake of vegetables allowed during VLCKD, and low-calorie laxatives.

### 2.5. Differences between Very Low-Calorie Ketogenic Diets with Meal Replacement or with Traditional Protein

According to the European Guidelines for the Management of Obesity in Adults, VLCKD includes proteins with a high biological value that are derived from milk, peas, whey, and soy [30]. This diet can be achieved by using meal replacement or natural foods [30]. Basciani et al. conducted a study comparing the effectiveness and safety of VLCKD for 45 days using whey or vegetable protein meal replacement foods with conventional animal protein in a group of patients with obesity and insulin resistance [57]. The results showed that after 45 days of VLCKD, there was a significant reduction in initial body weight in both the whey protein and plant protein groups. Although the animal protein group also showed a reduction in body weight, it was not statistically significant. The animal protein group also showed an increase in blood urea nitrogen and uric acid and a significant reduction in the estimated glomerular filtration rate compared to baseline values. The authors concluded that VLCKD based on whey or vegetable protein is a safer option than animal protein for patients with obesity [57]. Therefore, a VLCKD with whey and vegetable protein-based meal replacements is a more suitable option for these patients.

Based on scientific evidence, it is recommended to use meal replacements during the initial active ketogenic phase of a VLCKD to ensure a safe, effective, and controlled administration [57,58]. In fact, with the use of single-portioned meal replacement meals, the calibration of the diet is more accurate, and the content of calories, macronutrients, and micronutrients needed by the patient can be set more precisely and individually. Therefore, it would seem more appropriate to set up a VLCKD protocol with the use of meal replacements to determine greater safety, efficacy, and compliance prior to BS, with preference given to freeze-dried meal replacements, which generally have a higher protein content and lower fat and carbohydrate content. This would ensure a higher degree of weight loss and a better adherence, which is critical when considering of the short duration of the protocol.

## 3. Bariatric Surgery

### 3.1. Sleeve Gastrectomy

SG was conceived as the first surgical stage of biliopancreatic diversion with duodenal switch producing a malabsorptive and restrictive bariatric procedure [59]. When Regan et al. reported the results of SG as a first-stage procedure before RYGB in patients with BMI > 60 kg/m^2^ showing a mean weight loss of 37 kg and a mean BMI decrease of 13 kg/m^2^ after 11 months of follow-up, SG gained increasing interest as a stand-alone bariatric procedure [60]. Today, SG represents the most performed BS worldwide and further innovations, such as the use of the laparoscopy, changes in the surgical techniques, and the use of natural transluminal orifice endoscopic surgery, have been put in place to improve its outcomes [61].

Laparoscopic SG comprises a subtotal vertical gastrectomy, creating a tubular duct along the lesser curve with pylorus preservation [62]. Being considered quicker and easier to perform as it does not include any intestinal anastomosis, compared to other more complex bariatric procedures, its wide diffusion and acceptance also depends on the favourable outcomes reported in terms of weight loss, the reduction in obesity-related comorbidities, and the low rate of postoperative complications. SG does not only work as a restrictive procedure, but it provides important hormonal changes involving GLP-1, peptide YY (PYY), and ghrelin and leptin pathways, accounting not only for the several metabolic changes but also for the sharp decrease in feelings of hunger [62]. A review of the literature by Diamantis et al. revealed the percentage of excess weight loss to be 62.3%, 53.8%, 43%, and 54.8% at 5, 6, 7, and 8 or more years of follow-up, respectively [63]. Concerning T2DM, the research by Madadi et al. involving 2480 patients who underwent SG, the remission rate was 56.29% after 1 year follow-up [64]. However, the literature concerning long-term outcomes after SG alone or compared to other procedures is poor and disparate. Han et al. conducted a meta-analysis encompassing 2917 patients from randomized prospective and retrospective studies, which highlighted no differences in mid- and long-term weight loss between SG and RYGB; moreover, no difference in long-term T2DM remission was found [65]. On the other hand, a meta-analysis from Gu et al. reported the superiority of RYGB in T2DM remission at 3 years follow-up and in the percentage of excess weight loss and remission of T2DM, hypertension, and dyslipidaemia [66]. Furthermore, another meta-analysis by Lee et al. showed the superiority of RYGB in 1 and 3 years BMI loss and 1 and 5 years dyslipidaemia remission, but no differences were found in T2DM and hypertension remission compared to SG [67]. While the latter evaluated only early (<30 days) postoperative complication rates, reporting no differences between RYGB and SG [67], the research by Han et al. highlighted higher early postoperative complications (RR: 2.14) and reoperation (RR: 1.73) risks for RYGB and no difference in terms of late (≥30 days) postoperative morbidity [65].

Although SG is a safe procedure, burdened by low postoperative morbidity and negligible mortality, postoperative gastroesophageal reflux disease (GERD) represents a common issue for patients undergoing this procedure. The physiopathology of GERD has not been completely elucidated and different causes, such as increased intragastric pressure, reduced gastric emptying, and decreased lower oesophageal sphincter pressure, have been evocated [66,67]. The study by Yeung et al., involving 10,718 patients, showed a 23% rate of de novo GERD after SG, which is associated with a 28% and 8% rate of long-term esophagitis and Barrett’s oesophagus prevalence, respectively; in addition, GERD was the reason for conversion to RYGB in 4% of patients [68]. Weight regain represents another major drawback of SG. A recent meta-analysis including studies with long follow-up after SG showed a 27.8% rate of weight recidivism and a 19.9% rate of subsequent revisional rate [69].

SG is currently surgeons’ most preferred bariatric procedure due to its simplicity, the low related morbidity, and the good short- and mid-term weight loss and results regarding obesity-related comorbidities. However, its long-term reliability is uncertain, and GERD represents a major cause of discomfort and morbidity for individuals with SG.

### 3.2. Roux-en-Y Gastric Bypass

For many decades, RYGB represented the most frequently used bariatric procedure performed before being recently superseded by SG [70]. It consists of the creation of a small gastric pouch that is separated from the gastric remnant, the anastomosis of the gastric pouch to the distal part of a transected bowel loop (Roux-en-Y limb), and the connection of the proximal part of the transected small bowel loop (biliopancreatic limb) to the Roux limb at a previously defined distance from its anastomosis with the gastric pouch; many different methods of reproducing this anatomical construction have been described [71]. The aim of this reconstruction is to combine the restrictive effect of a tiny gastric pouch to the malabsorption occurring in the common alimentary and biliopancreatic limb length [71]. Once thought to be the most relevant mechanism to determine weight loss after RYGB, the recent literature has showed how no changes in carbohydrate and protein absorption and only low fat malabsorption after proximal RYGB with an estimated 11% contribution on total postoperative weight loss in the early period due to the malabsorptive phenomenon [72]. However, recent studies focusing on mid- and long-term weight loss after RYGB has showed encouraging results supporting its employment [73,74,75]. Golzarand et al. reported the percentage of excess weight loss being 62.58% after 5 years and 63.52% after 10 years in 1671 patients who underwent RYGB [73]. Similar results were outlined by O’Brien et al., with 55.4% of excess weight loss after 10 years or more from BS [74]. Concerning obesity-associated medical problems, RYGB has also been demonstrated to be effective [75]. Compared to medical treatment, RYGB has been revealed to be superior in terms of T2DM remission (OR: 76.37) and patients after RYGB showed significantly inferior serum levels of HbA1c, triglyceride, low-density lipoprotein cholesterol, and systolic blood pressure [75].

On the other hand, RYGB is characterized by some drawbacks which are still debated. Although RYGB showed better results in long-lasting T2DM remission compared to SG, T2DM relapse after 10 or more years follow-up is estimated to be 30% [76]. Furthermore, RYGB may be badly tolerated due to the occurrence of nutritional issues. Post-RYGB anaemia can reach 45–50% incidence as a consequence of iron and B12 vitamin deficiency; hypoproteinaemia has a 10–15% incidence and mineral deficiency is also frequent [77]. In the end, in contrast with the short- and mid-term results of optimal weight loss, long-term weight regain after RYGB is documented in 20–35% of patients [78].

### 3.3. One-Anastomosis Gastric Bypass

OAGB consists in producing a small size gastric pouch on a 36 Fr bougie with a single anastomosis with the small bowel at 150–200 cm from the Treitz ligament [79]. With this anatomical reconstruction, the restrictive and malabsorptive principia of RYGB are conserved with only one anastomosis, reducing surgical complexity and the sources of postoperative complications at the same time [79]. The IFSO published an update position statement on OAGB analysing the results of all the literature on this procedure [80]. Short-term results in terms of weight loss are encouraging. Nine RCTs with 501 patients who underwent OAGB showed a global percentage of excess weight loss of 67.85% and 87.54% excessive BMI loss after 25.33 months of mean follow-up. Concerning associated medical problems, patients who underwent OAGB showed positive T2DM, obstructive sleep apnoea syndrome (OSAS), hypertension, and dyslipidaemia remission rates [80]. Although OAGB is widely carried out, as it considered effective, easy, and quick to perform and has a low postoperative complication rate [81], some nutritional and malabsorptive issues must be considered [82,83]. A comparative systemic review and meta-analysis by Tourky et al. showed a significantly increased percentage of excess weight loss and percentage of total body weight loss at 3-year follow-up but also highlighted an increased risk of postoperative malnutrition (OR: 3) and hypoalbuminemia (OR: 2.38) for OAGB compared to RYGB [83]. Moreover, the anatomical reconstruction of OAGB theoretically exposes the patients to an increased incidence of bile reflux, which can cause esophagitis and is a potential risk factor for oesophageal cancer; postoperative bile reflux incidence varies between studies from 7.8 to 55.5% [82]. In the end, long-term postoperative outcomes after OAGB are still not well documented in the literature with only few retrospective studies reporting a 10-year or more follow-up.

### 3.4. Single-Anastomosis Duodeno-Ileal Bypass

SADI consists of a gastric greater curvature resection, followed by a resection of the duodenum 3–4 cm from the pylorus, and then a duodeno-ileal anastomosis is performed, producing a 200 cm efferent limb [84].

As it has only recently been proposed and adopted, research evaluating outcomes after SADI is limited, especially when considering long-term results. A comparative systematic review and meta-analysis by Verhoeff et al. evaluated 3319 patients who underwent a malabsorptive procedure, including 1704 patients receiving SADI [85]. They reported a significantly shorter operative time and length of stay and postoperative complication rate for SADI. In addition, no differences in terms of weight loss, associated medical problems remission, and nutritional deficiencies were highlighted; however, follow-up in the included studies was too short to produce solid conclusions and, although subgroup analysis was performed, the reliability of the results of this meta-analysis was affected by the heterogeneity of the comparative group, which included patients who underwent different malabsorptive procedures [85]. Sanchez-Pernaute et al. published their 10-year follow-up case series of 123 SADI, showing 80% and 34% of excess weight loss and total body weight loss, respectively; a total of 12 of 41 diabetic patients needed insulin treatment at the end of follow-up and 12 of 123 had undergone revisional surgery due to chronic hypoproteinaemia [86].

SADI has also demonstrated encouraging results as revisional surgery after failure of previous restrictive procedures [87], but long, high-quality follow-up studies are needed to evaluate long-term efficacy of this procedure in a primary and revisional setting.

### 3.5. Perioperative Issues

#### 3.5.1. Perioperative Technical Issues

Individuals undergoing BS represent a peculiar population with their own specific characteristics that make each surgical step insidious. As a matter of fact, considering the two most common bariatric procedures performed worldwide, the learning curve threshold has been shown to be set at 100–200 laparoscopic SG and up to 500 laparoscopic RYGB for the single surgeon to master these procedures [88].

#### 3.5.2. Port Placement

Port placement is the first step of any laparoscopic surgery. Hasson’s technique, conceived in 1971, consists of performing a mini-laparotomy to gain access to the peritoneal cavity and place the optic trocar under direct visualization to avoid inadvertent abdominal organ injuries [89]. This technique, which was introduced as an alternative to blind trocar placement to reduce procedure-associated complications, is often impossible to realize as a consequence of the abdominal wall thickness in bariatric patients, although a “large” mini-laparotomy is performed with the successive risk of CO_2_ leakage that can compromise the surgical performance [90]. Access to the abdominal cavity is challenging not only due to abdominal wall thickness but also because individuals with obesity, especially females, have a high dense abdominal barrier and thick peritoneum [90]. Moreover, the umbilicus in this population can have variable positions and the bariatric surgeon has to use different landmarks to avoid optic port placement in a position that can affect surgical performance [91,92].

Closed techniques, such as direct trocar insertion (DTI) and the Veress technique, are commonly used in individuals with obesity, but they are not riskless, as they consist in the blind insertion of a sharp instrument into the abdomen [93,94]. Two randomized clinical trials have been performed comparing these access techniques in individuals with obesity [93,94]. Ertugrul et al. reported two major complications in the DTI group vs. no major complications in the Veress group in 81 patients scheduled for bariatric laparoscopic surgery using a bladed trocar for the DTI technique; abdominal access was faster in the DTI group while no difference in terms of access failure rate was found [93]. Similar findings were reported by Ikechebelu et al. in 135 women with obesity undergoing diagnostic laparoscopy for infertility—the only difference resulting from the two groups was faster access time to the abdominal cavity in favour of the DTI group [94]. At present, no recommendation has been developed regarding which technique is the most safe and feasible and any bariatric surgeon should be comfortable with multiple techniques.

#### 3.5.3. Patient-Related and Intraoperative Factors

How anatomical and intraoperative factors can affect the complexity of bariatric procedures is a debated topic, as most surgeons agree that some specific features have a relevant impact, but the current literature is very scant regarding this issue. A worldwide international survey based on 370 expert bariatric surgeons was performed by Shahabi et al., which focused on how many anatomical and intraoperative factors could make the procedure easier or more complicated [92]. Some anatomical features, such as hepatomegaly, a large sized hiatal hernia, a thick falciform ligament, and a thick omentum, were considered as moderately or highly complicating to the bariatric procedure. As a matter of fact, the aforementioned characteristics play a part in reducing the accessibility to the stomach and the bowel for resecting, stapling, and suturing and make it more difficult to achieve a correct operative position. Consequently, the higher is the patient’s BMI, the harder the operation is expected to be. A total of 39.7% of the experts surveyed agreed that a BMI > 50 kg/m^2^ makes the performance of operations moderately difficult and 10.8% thought that it makes the procedure very difficult; a BMI > 60 kg/m^2^ makes the operation very difficult for 34.3% of experts and extremely difficult for 12.1%. These data should, however, be taken carefully, as the distribution of adipose tissue determines surgical difficulty to a greater extent than BMI and patients’ phenotype, i.e., gynoid vs. android, may play a pivotal role in determining surgical difficulty. Indeed, some individuals with a BMI > 50 kg/m^2^ may present with peripheral obesity (gynoid phenotype) and be easy to operate on, while on the other hand an individual with central obesity and a BMI between 35 and 40 kg/m^2^, may be very challenging to operate on because the presence of most of the fat in the abdomen hinders the possibility of obtaining enough room with the pneumoperitoneum to perform the bariatric procedure with ease. Liver cirrhosis, which is not rarely associated because of progressive non-alcoholic steatohepatitis, also represents an unfavourable characteristic and 32.4% of the surgeons surveyed affirmed that it makes the operation moderately difficult, while 21.8% declared that it makes the procedure very difficult [92].

#### 3.5.4. Anaesthesia

Individuals with obesity represent a significant challenge for anaesthesiologists, as obesity and its related medical issues deeply affect bariatric perioperative management. With the current obesity epidemic, the literature concerning the pitfalls of anaesthesia in this specific population is progressively developing.

#### 3.5.5. Perfusion

Providing one or more venous access is the first step in preparing the patient for anaesthesia. Intravenous cannulation can sometimes be difficult due to different factors. Obesity is commonly considered a complicating condition as it affects vein palpation and visualization [95]. The research by Brandt et al. showed that a higher BMI is associated with the absence of clinically detectable veins and that ultrasonography guarantees 100% success in finding a peripheral vein suitable for cannulation [95].

#### 3.5.6. Intubation

Endotracheal intubation is commonly considered to be more difficult in patients with obesity; however, there is no clear evidence that difficult intubation is more frequent than in lean populations [96]. A large French cohort study reported an increased incidence of failed primary intubation and of difficult intubation in individuals with obesity compared to individuals without obesity, although the factors related to an increased risk of failed intubation did not differ from those seen in the normal weight population (Mallampati III/IV grade, cervical spine rigidity, OSAS) [97,98]. Bariatric patients frequently have an increased neck circumference and a neck circumference/thyromental distance ratio due to fat distribution which are associated with an increased Mallampati grade [99]. Moreover, the frequent association between obesity and diabetes adds another factor that can complicate the intubation, as it has been demonstrated that diabetic patients suffer from increased osteoarticular stiffness, which provokes cervical spine rigidity and reduction in consented motions during intubation [100]. A history of OSAS should always be investigated preoperatively as its incidence is elevated in the morbidly obese population (35–93%) and it can affect many aspects of anaesthesiologic perioperative management [101]. Videolaringoscopy could be employed to ease difficult intubations; however, there is still weak evidence regarding its actual benefits in this specific situation. On the other hand, preferring a ramped position to a flat supine position at the moment of induction and intubation eases the procedure [101].

#### 3.5.7. Ventilation

Up to 20% of patients with morbid obesity are diagnosed with Obesity Hypoventilation Syndrome, which is defined as the coexistence of BMI ≥ 30 kg/m^2^ and daytime hypercapnia with PaCO_2_ > 45 mmHg during wakefulness in the absence of an alternative neuromuscular, mechanical, or metabolic explanation for hypoventilation, and its incidence reaches 50% in patients with BMI ≥ 50 kg/m^2^ [102]. Different mechanisms are implicated: the obesity-related restrictive respiratory mechanic, the central respiratory drive depression determined by chronic hypercapnia and the consequently increased bicarbonate retention, and leptin resistance [103]. In these patients, bilateral pulmonary atelectasis frequently coexists, reducing respiratory reserve [103]. Obesity Hypoventilation Syndrome exposes the bariatric patient to perioperative desaturation and to an increased risk of respiratory complications, depending on the difficulty of reaching a balance between adequate oxygenation and the risk of pulmonary barotrauma [104]. In addition, the concurrence of OSAS, which delineates the so-called overlap syndrome, further accentuates the aforementioned issues. Moreover, the need to set the patient in a Trendellenburg position during certain technical surgical steps increases the pressure performed by the abdomen on the chest furtherly reducing respiratory volumes and reserves. Intraoperative pressure-controlled ventilation with low tidal volume, carefully titrated positive end-expiratory pressure, and lung recruitment manoeuvres result in better intraoperative oxygenation, atelectasis mitigation, and reduced postoperative respiratory complications after laparoscopic BS [104].

#### 3.5.8. Extubation

The respiratory function alterations induced by obesity and its related medical issues in combination with the effects of anaesthetic drugs also exposes the bariatric patient to an increased risk of respiratory insufficiency from the moment of extubation [105]. To help avoid respiratory complications, bariatric patients should be as awake as possible prior to extubating in the operating room [105]. To achieve this, many attempts to modify anaesthetic drugs protocols have been carried out. Patients undergoing BS have different pharmacokinetics compared to leaner populations, as liposoluble drugs are stocked in fat tissue, releasing them slowly, which in turn may create long-lasting residual effects [106]. Avoiding opioids or using short-acting opioids along with adjuvants and avoiding or minimizing the need for neuromuscular blocking agents can directly cut down the number of postoperative pulmonary complications in patients with obesity [106].

### 3.6. Very Low-Calorie Ketogenic Diet in Bariatric Surgery

#### 3.6.1. The Use of Ketogenic Diet on Patients with Obesity Scheduled for Bariatric Surgery

BS is known to be the most effective and durable therapeutic means for the long-term treatment of morbid obesity [107]. Currently, laparoscopic surgery is the preferred method for BS in almost all cases. Patients who require BS often have a steatotic liver, which can make the surgery technically challenging [107]. This can lead to longer surgery times, an increased risk of bleeding during surgery, anastomotic complications, and in some cases, suboptimal bariatric anatomy, which can compromise long-term results [108,109]. Another challenge during bariatric surgery is increased intra-abdominal fat, especially in patients with central obesity. This can reduce the working space and make it difficult to expose anatomical landmarks, as well as impair complex surgical tasks, such as knotting and suturing [13,110,111]. Therefore, preoperative interventions to reduce body weight, hepatomegaly, and intra-abdominal fat before laparoscopic bariatric surgery could benefit both surgeons and patients by reducing surgical risk [13,110,111]. However, there is no clear consensus on the most effective dietary approach.

KDs have been used as a therapy for epilepsy since the 1920s and have been widely used for obesity treatment since the 1960s [112]. These diets are characterized by a high intake of fats and proteins, with a significant reduction in carbohydrate consumption, inducing a state of physiological ketosis [113]. For instance, a VLCKD involves a drastic reduction in carbohydrate intake (less than 50 g per day, providing about 13% of caloric intake), with adequate protein intake (about 0.8–1.2 g per kg of ideal body weight, providing about 45% of caloric intake) and a relatively high intake of fats (approximately 42% of caloric intake), with an average energy intake of 800 kcal per day [113]. Strong and supportive evidence suggests that KDs are effective for weight loss therapy, and they may be a valid option for patients at higher risk who need to achieve rapid weight loss [45,114]. Patients often report satisfaction with this nutritional approach, possibly due to the anorexigenic, euphoric, and mood-stabilizing effects of ketone bodies, which reduce hunger and promote a feeling of rapid satiety [112].

One of the first study addressing the effect of VLCKD on patients with obesity scheduled for BS was performed by Leonetti et al. [48]. The study evaluated the efficacy of a sequential diet regimen called OPOD, in 50 patients with a mean BMI of 53.5 ± 8.4 kg/m^2^, with and without T2DM, who were scheduled for laparoscopic BS. The OPOD regimen consisted of a 10-day KD (600 kcal per day, 15 g of carbohydrates, 80 g of proteins, and 23 g of lipids), followed by a 10-day VLCD (800 kcal per day, 55 g carbohydrates, same proteins, and 30 g lipids), and finally an LCD (1100 kcal per day, with an increase in carbohydrates up to 145 g, 60 g proteins, and 33 g lipids) until the surgery. The OPOD regimen scheme used by Leonetti et al. is reported in Table 1.

The patients in the study were assessed at baseline (T0) and after 10 days (T1), 20 days (T2), and 30 days (T3). The results showed that body weight, BMI, waist circumference, and neck circumference were significantly lower at T1, T2, and T3 than at T0 in the 48 patients who completed the OPOD regimen. Additionally, in patients with T2DM, fasting plasma glucose levels decreased significantly, allowing for a reduction in diabetic medications. The study concluded that the OPOD, which includes 10 days of VLCKD, was safe and effective for patients with obesity with or without T2DM who were candidates for BS [48]. Similarly, Albanese et al. aimed to compare surgical outcomes and weight loss in two groups of patients who were offered two different pre-operative diets: VLCD and VLCKD. The study involved 178 patients, with VLCKD implemented for 72 patients and VLCD implemented for 106 patients. The mean age was 43 years, and the mean BMI before the diet was 46.3 ± 6.3 kg/m^2^ for the VLCKD group and 43.1 ± 6.9 kg/m^2^ for the VLCD group. The results showed that absolute weight loss was significantly better in the VLCKD group than in the VLCD group (5.8 ± 2.4 vs. 4.8 ± 2.5 kg; *p* = 0.008), while there were no significant differences in excess BMI loss (10.4 ± 4.0 vs. 10.0 ± 5.6%; *p* = 0.658). The VLCKD regimen consisted of 1.4 g of protein per kg of ideal body weight, <20–30 g of carbohydrates, and 15–20 g of lipids per day, divided into three main meals with a maximum caloric intake of 700 kcal per day. Breakfast and dinner were replaced by a diluted powder containing whey proteins enriched with amino acids, while lunch included animal or plant-derived protein natural food and 200 g of vegetables. The study recommended the integration of trace elements diluted in 2 L of water per day [36]. The VLKCD scheme used by Albanese et al. is reported in Table 2.

While in the study by Albanese et al., VLCKD was developed using regular food, Pilone et al. proposed a sequential diet regimen consisting of a VLCKD for 10 days (referred to as the V-diet), followed by a hypocaloric scheme for the next 20 days (referred to as V-hypo), with a gradual increase in caloric intake [115]. Pilone et al. proposed a dedicated KetoStationkit for use during the first 10 days of the regimen, along with a hypocaloric scheme for the next 20 days. The KetoStationkit included a protein powder (82 g of protein from whey and caseinate for every 100 g of product) and nutritional supplements (multiminerals, multivitamins, and omega 3 fatty acids). During the V-diet, patients were advised to consume eight scoops of ketogenic powder per day for females and nine scoops per day for males, with each scoop diluted in 100–200 mL of water (one scoop containing 10 g, including 0.3 g of carbohydrate, 8.2 g of protein, and 0.4 g of fat). Patients could add vegetables to their regimen during lunch and dinner and were encouraged to consume at least 2 L of fluids per day. Ketone body levels were measured in the plasma and urine, and routine laboratory tests and anthropometric measurements were conducted at enrolment (T0), after 10 days (T1), and after 30 days (T2). The results of the study showed a significant decrease in body weight, BMI, and waist circumference at T0 and T1, T0 and T2, and T1 and T2 (*p* < 0.05). Additionally, a bioelectrical impedance assay showed a significant reduction in visceral fat at T1 and T2. The study also observed a significant improvement in several clinical parameters, including glycaemic and lipid profile parameters, associated with a mean 30% reduction in liver volume. The study concluded that a VLCKD performed using a dedicated KetoStationkit was safe and effective in reducing weight and liver volume in patients with obesity who were candidates for BS [115].

Furthermore, Schiavo et al. investigated the clinical impact of a micronutrient-enriched ketogenic diet on patients with obesity who were candidates for BS [50]. The study involved a 4-week preoperative period during which the patients adhered to a ketogenic food plan, providing approximately 1200 calories per day, consisting of 4% carbohydrates, 71% fats, and 25% proteins. The food plan was supplemented with a composition of nutrients (Ketocompleat, MVMedical Solutions, Serravalle, Repubblica San Marino) [50].

An example of the preoperative KD daily plan used by Schiavo et al. is reported in Table 3.

All subjects obtained a significant reduction in body weight (males 10.3%, *p* < 0.001 and females 8.2%, *p* < 0.001) and in left hepatic lobe volume (−19.8%; 503 ± 61 cm^3^ vs. 627 ± 85 cm^3^, *p* < 0.001) [50]. Furthermore, Schiavo et al., with the aim to prospectively compare the effects on weight loss, fat mass, fat free mass, and resting metabolic rate in two groups of patients with obesity scheduled for BS and who were randomized to two different diets (LCKD diet vs. LCD) after intragastric balloon placement, showed that the LCKD group displayed a significantly lower decrease in fat free mass and resting metabolic rate when compared with the LCD group (3.55 vs. 14.3%, *p* < 0.001; 9.79 vs. 11.4%, *p* < 0.001, respectively) [116]. Moreover, fat mass decreased more significantly with LCKD compared to LCD (41.6 vs. 33.1%, *p* = 0.0606). The authors concluded that, based on their findings, they were able to support the hypothesis that LCKD is associated with an increased fat mass loss while reducing the fat free mass loss and the resting metabolic rate [116]. In addition, in another study, Schiavo et al. were able to show in a pilot, prospective, randomized multicentre comparative study that LCKD associated with continuous positive airway pressure was able to alleviate OSAS in patients with obesity scheduled for bariatric/metabolic surgery [117].

#### 3.6.2. Assessment of Surgical Outcomes

Many bariatric surgeons suggest an aggressive weight reduction regimen to patients before undergoing BS, as preoperative weight loss may improve patient outcomes. Some surgeons may even withhold surgery if a certain threshold of preoperative weight loss is not achieved, although the scientific evidence supporting this practice is unclear. However, in an effort to improve patient outcomes after bariatric procedures, many now insist that patients meet preoperative weight loss goals before undergoing surgery. The Canadian Clinical Guidelines recommend a preoperative weight loss of 10% of body weight within 6 months through dietary modification [118], while some insurance companies in the United States require a 5–10% preoperative weight loss and the attendance of multiple nutritionist consultations before surgery approval [119]. The purported benefits of preoperative weight loss include selecting the most motivated patients, acclimating patients to restricted intake, reducing perioperative morbidity, and decreasing liver volume, leading to shorter operative times [120]. However, the National Institutes of Health consensus statement does not mandate preoperative weight loss but rather evaluates patients based on BMI, co-morbidities, and previous weight loss attempts, without considering successful preoperative weight loss [121].

Bariatric surgeons commonly believe that weight loss before BS leads to technically simpler procedures. However, the evidence for mandatory preoperative weight reduction is limited and conflicting. While reducing liver volume and intra-abdominal fat may make surgery easier and decrease co-morbidities, this hypothesis has not been definitively established. The systematic review of 17 trials, encompassing approximately 4611 patients, found preoperative weight loss to be beneficial, while 10 studies, encompassing 2075 patients, found no benefit [45]. Laparoscopic RYGB patients who underwent preoperative weight loss experienced a 12.5-min shorter operative time. In terms of postoperative weight loss, nine studies (39%) reported a positive correlation, while fifteen (62.5%) reported no benefit. Nine studies reporting perioperative complications (852 patients) revealed no difference in complication rates, while two studies (1234 patients) suggested a significant decrease associated with preoperative weight loss [45]. Therefore, a large-scale, multicentre, randomized, controlled trial with sufficient power is necessary to determine the effectiveness of preoperative weight loss.

Up to this point, it has been difficult to determine whether the results of weight loss before BS are solely due to weight loss or whether a specific KD provides additional benefits. Albanese et al. sought to answer this question by comparing weight loss and surgical outcomes in two groups of patients who followed different diets for three weeks before surgery: a VLCKD and a VLCD [36]. A total of 178 patients were enrolled in the study, with 72 following VLCKD and 106 following VLCD. While both groups were informed that weight loss before surgery was mandatory, the patients’ preferences influenced the type of diet they followed. After three weeks, the VLCKD group had a better absolute weight loss than the VLCD group (5.8 ± 2.4 kg vs. 4.8 ± 2.5 kg, *p* = 0.008), but there was no significant difference in the percentage of excess BMI loss (respectively, 10.4 ± 4.0% and 10.0 ± 5.6%, *p* = 0.658). All patients underwent laparoscopic SG. While the mean operative times and hospital stays were comparable in both groups, the VLCKD group had lower drainage output (141.2 ± 72.8 mL vs. 190.7 ± 183.6 mL, *p* = 0.032), higher post-operative haemoglobin levels (13.1 ± 1.2 mg/dL vs. 12.7 ± 1.5 mg/dL, *p* = 0.04), and a lower percentage of patients requiring prolonged hospital stays (2.8% vs. 10.4%, *p* = 0.048) compared to the VLCD group. The authors concluded that the advantages of VLCKD were not strictly related to surgical manoeuvres, as the operative time was comparable between the two groups but rather to a better metabolic and nutritional status that positively influenced tissue healing [36].

Table 4 summarises the main findings of studies on KDs before BS.

## 4. Conclusions

Weight loss before BS is crucial for patients, as it leads to various benefits, such as a decrease in liver volume and visceral fat, a lower risk of intra- and post-operative complications, shorter surgery times, and reduced hospital stays. VLCKDs have proven to be a safe and effective way to achieve weight loss and may be considered as an option in the pre-operative period of BS. However, larger RCTs with well-defined dietary protocols are necessary to make definitive conclusions. Additionally, a longer follow-up period is needed to evaluate the long-term effects of preoperative weight loss.

## Figures and Tables

**Table 1:** The obese preoperative diet (OPOD) [48].

Regimen OPOD		
VLCKD (Days 1–10)	VLCD (Days 11–20)	LCD (Days 21–30)
Take 8–9 * scoops (one scoop = 10 g; 0.3 g of carbohydrates; 8.2 g of protein; 0.4 g of fats) of ketogenic powder per day each diluted in 100–200 mL of water and oral supplements as follows: Breakfast—two scoops and two tablets of multimineral. Lunch—two scoops and two tablets of multivitamins. Dinner—two scoops and two tablets of omega 3 Snacks (mid-morning, mid-afternoon, after dinner)—one scoop. Drink each day at least 2 L of liquids (except sweetened drinks). Limit physical activity and excessive stress. Free consumption of vegetables at lunch and dinner (minimum 500 g day). Allowed—20 g of extra virgin olive oil per day. Daily energy intake 560–595 Kcal: ✓ Carbohydrates: 15 g, ✓ Proteins: 72–80 g, ✓ Lipids: 23–24 g.	Stop VLCKD treatment and oral supplements. Start a very low-calorie diet as follows: Breakfast: Recommended food—200 g of semi- skimmed milk or low-fat yogurt or unsweetened orange juice, 20 g of rusks or 20 g of bread. Forbidden food—sweets or brioches. Lunch—150 g of lean meat or 200 g of fish, free consumption of vegetables (minimum 250 g), 100 g of fruit. Dinner—100 g of low-fat cheese, free consumption of vegetables (minimum 250 g), 100 g of fruit. Allowed—20 g of extra virgin olive oil per day. Daily energy intake 810 Kcal: ✓ Carbohydrates 55 g, ✓ Proteins 80 g, ✓ Lipids 30 g.	Increase the amount of carbohydrates as the following scheme: Breakfast: Recommended food—200 g of semi- skimmed milk, or low-fat yogurt or unsweetened orange juice, 40 g of rusks or 50 g of bread. Forbidden sweets or brioches. Lunch—80 g of pasta or bread, free consumption of vegetables (minimum 250 g), 100 g of fruit. Dinner—150 g of lean meat or 200 g of fish or 100 g of low-fat cheese, free consumption of vegetables (minimum 250 g), 100 g of fruit. Allowed—20 g of extra virgin olive oil per day. Daily energy intake 1100 Kcal: ✓ Carbohydrates 145 g, ✓ Proteins 60 g, ✓ Lipids 33 g.

* Eight for females, nine for males. OPOD, obese preoperative diet; VLCKD, very low-calorie ketogenic diet; VLCD, very low-calorie diet; LCD, low-calorie diet.

**Table 2:** Very low-calorie ketogenic diet scheme used by Albanese et al. [36].

Meal	
Breakfast	Two measuring cups of protein powder in water or yogurt with a fat content of 0.1% (either plain or fruit-flavoured). Coffee is also acceptable.
Lunch*	A total of 180 g of animal proteins (such as beef, calf, rabbit meat, chicken, or turkey breast) or 200 g of fish proteins (such as anchovies, sardines, tuna, mackerel, lobster, shrimps, pike, cod, rhombus, sole, sea bass, grouper, snapper, sea bream, cuttlefish, squid, octopus, salmon, or swordfish) or 200 g of plant-based proteins (such as tofu, seitan, or tempeh), along with 200 g of vegetables (such as chard, chicory, zucchini, cauliflower, fennel, eggplant, broccoli, lettuce, radish, artichoke, or spinach).
Dinner *	Four measuring cups of protein powder in water or yogurt with a fat content of 0.1% (either plain or fruit-flavoured), along with 200 g of vegetables (such as chard, chicory, zucchini, cauliflower, fennel, eggplant, broccoli, lettuce, radish, artichoke, or spinach).

* the consumption of two small scoops of olive oil per day is allowed, but vinegar is not permitted.

**Table 3:** An example of preoperative ketogenic diet daily plan used by Schiavo et al. [50].

Meal	
Breakfast	Egg (100 g), salt (0.13 g), pepper (0.033 g), olive oil (5 g)
Snack	Nuts (30 g)
Lunch	Lamb loin 145 g), olive oil (10 g), salt (1.5 g), pepper (0.13 g), asparagus (143 g)
Snack	Cheddar cheese (30 g)
Dinner	Ketocompleat (40 g), water (250 mL)
Total calories 1215.4 kcal: ✓ Fat: 71% (96.1 g), ✓ Carbs: 4% (14.2 g), ✓ Protein: 25% (76 g).	

**Table 4:** Main findings of studies on ketogenic diet before bariatric surgery.

Reference	Population	Aim and Intervention	Findings
Leonetti et al. [108]	19 M; 31 F	Assessment of the effectiveness of a sequential diet regimen termed the OPOD in morbidly obese patients with and without type 2 diabetes mellitus scheduled for bariatric surgery. OPOD regimen: VLCKD for 10 days; VLCD for 10 days; LCD for 10 days.	Reduction in body weight, body mass index, waist circumference, and neck circumference; amelioration in fasting plasma glucose levels; reduction in liver volume; and improvement of liver steatosis.
Albanese et al. [30]	39 M; 139 F	Compared surgical outcome and weight loss in two groups of patients who were offered two different pre-operative diets: VLCD and VLCKD: 72 patients followed a pre-operative VLCKD and 106 a VLCD.	Absolute weight loss was significantly better in the VLCKD than in the VLCD group, while no significant differences were observed in % of excess body mass index loss. VLCKD showed better results than VLCD on surgical outcome, influencing drainage output, post-operative haemoglobin levels, and hospital stay.
Pilone et al. [109]	44 M; 75 F	Evaluation of safety, efficacy, and acceptability of a VLCKD in patients before bariatric surgery using a sequential diet regimen: VLCKD for 10 days, followed by a hypocaloric scheme for 20 days, with the progressive recovery of calorie levels.	Weight, body mass index, waist circumference, and visceral fat decreased significantly. Furthermore, a significant improvement in several clinical parameters, including liver volume and glycaemic and lipid profile parameters were observed. The majority of patients declared themselves satisfied or very satisfied. The adverse effects were mild, of short duration, and not clinically relevant.
Schiavo et al. [110]	10 M; 17 F	To assess the safety and the effectiveness of a 4-week preoperative KMED in reducing body weight and left hepatic lobe volume in patients scheduled for bariatric surgery. Ketogenic food plan (from 1150 to 1250 kcal/day) consisted of 4% carbohydrates, 71% fats, and 25% proteins. Dinner was substituted by Ketocompleat (MVMedical Solutions, Serravalle, Repubblica San Marino). Ketocompleat is a supplement included on the register of food supplements of the Italian Minister of Health (code number 94721), and due to its carbohydrate-free formulation, may be associated to a low-carbohydrate ketogenic diet.	The study indicates that a 4-week preoperative KMED is safe and effective in reducing body weight and left hepatic lobe volume in patients with obesity scheduled for bariatric surgery.
Schiavo et al. [111]	22 M; 26 F	To prospectively compare the effects on weight loss, fat mass, fat-free mass, and resting metabolic rate in two groups of patients who were randomized to two different diets: LCKD and a standard LCD after intragastric balloon placement. The macronutrients composition of the LCD and LCKD was 40% carbohydrates, 43% proteins, and 15% fats (~ 1200 kcal/day) and 4% carbohydrates, 25% proteins, and 71% fats (~ 1200 kcal/day), respectively.	The LCKD group showed a significantly lower decrease in free fat mass and resting metabolic rate when compared with the LCD group. Fat mass decreased more significantly with LCKD compared to LCD, without negative impact on renal function.
Schiavo et al. [112]	44 M; 26 F	To assess the clinical advantage of pre-bariatric surgery CPAP alone or in combination with a LCKD on apnoea–hypopnoea index and CRP levels in patients with obesity and obstructive sleep apnoea syndrome. The ketogenic food plan (from 1150 to 1250 kcal/day) consisted of 4% carbohydrates, 71% fats, and 25% proteins. Dinner was substituted by Ketocompleat (MVMedical Solutions, Serravalle, Repubblica San Marino)	Apnoea–hypopnea index scores improved significantly in both groups. Combining CPAP and LCKD registered no advantage on the apnoea–hypopnoea index score. Furthermore, CPAP + LCKD had a greater impact on CRP levels than CPAP alone demonstrating a positive impact on chronic inflammatory status.

OPOD, obese preoperative diet; VLCKD, very low-calorie ketogenic diet; VLCD, very low-calorie diet; LCD, low-calorie diet; MKED, ketogenic micronutrient-enriched diet; LCKD, low-calorie ketogenic diet; CPAP, continuous positive airway pressure; CRP, C reactive protein.

## Data Availability

No new data were created or analysed in this study. Data sharing is not applicable to this article.
